# Now I feel like I’m going to get to it soon: a brief, scalable intervention for state procrastination

**DOI:** 10.1186/s40359-025-03388-3

**Published:** 2025-10-16

**Authors:** Anusha Garg, Shivang Shelat, Jonathan W. Schooler

**Affiliations:** Department of Psychological and Brain Sciences, 251, University of California Santa Barbara, Santa Barbara, CA 93106 USA

**Keywords:** Procrastination, Temporal decision model, Intervention, Social desirability bias

## Abstract

Procrastination is a pervasive habit that undermines well-being, productivity, and mental health. Developing brief and scalable interventions to address this issue is crucial. Here, we tested the efficacy of an intervention grounded in the Temporal Decision Model of procrastination. Our intervention targeted state-level procrastination by simultaneously reducing task aversion and enhancing outcome utility. A total of 1,035 participants were randomly assigned to an experimental group or one of two control groups. The experimental group engaged in a structured activity that adapted an Affect Labeling technique with subtask generation/reward selection; the former stage aimed to reduce task aversion and the latter stage aimed to enhance outcome utility. Control groups responded to neutral or some task-related questions. Participants rated task aversion, outcome utility, mood, stress, motivation, and likelihood of completing the procrastinated task. Social desirability bias was measured and controlled for. The experimental group reported significantly higher task completion likelihood, greater outcome utility, improved mood, and a larger utility–aversion gap compared to control groups. Mediation analysis revealed that the utility–aversion gap and mood partially mediated the relationship between group assignment and task completion likelihood. These findings illustrate the potential effectiveness of a brief, low-effort intervention for reducing state procrastination. By enhancing outcome utility and employing structured strategies, this intervention suggests a scalable solution with potential applications in digital platforms and workplace settings to improve task engagement and productivity.

Procrastination, defined as the voluntary delay of an intended action despite anticipating negative consequences [48], is a pervasive and maladaptive behavior. This delay is consistently linked to diminished well-being, poorer mental health, impaired performance, and financial strain [28, 42, 51, 54], adversely affecting individuals' educational, professional, and personal domains [45]. Given its consequences, the development of effective interventions to mitigate procrastination is imperative.

Understanding the underlying mechanisms driving procrastination is crucial for designing effective interventions. There are three frameworks relevant to the current research that attempt to explain why individuals procrastinate on tasks: the emotion regulation perspective, the Temporal Motivation Theory, and the Temporal Decision Model. The emotion regulation perspective posits that individuals procrastinate to regulate negative emotions, engaging in mood-repair behaviors by avoiding aversive tasks in favor of immediately gratifying activities [5, 25, 47]. The greater the *task aversion* (i.e., perceived unpleasantness of a task), the more likely it is for individuals to delay task-relevant action to alleviate negative affect since they opt for short-term emotional relief over long-term goal pursuit [43, 44, 55, 56].

While the emotion regulation perspective offers an explanation for why individuals procrastinate, the Temporal Motivation Theory attempts to clarify why they eventually engage with the tasks they delay. The Temporal Motivation Theory [50] suggests that individuals engage in procrastinated tasks when the outcomes of completing the task are highly desirable. It posits that the *outcome utility* (i.e., perceived value or utility of task completion) increases over time, making task engagement more appealing closer to deadlines. As such, motivation to engage in a task intensifies as the deadline approaches.

Integrating the emotional regulation perspective with the Temporal Motivation Theory, the Temporal Decision Model conceptualizes procrastination as a cost–benefit analysis weighing task aversion against outcome utility. Individuals are more likely to postpone tasks when the immediate aversiveness outweighs perceived benefits. Although individuals often intend to act when tasks seem less aversive or more rewarding in the future, they typically delay until the outcome utility surpasses task aversion [62]. This dynamic interplay between task aversion and outcome utility provides groundwork for not only the development of a generalizable theory of procrastination, but also for creating interventions that target both task aversion and outcome utility to reduce procrastination.

The TDM primarily emphasizes task-specific cost–benefit evaluations, in which procrastination arises when perceived aversiveness outweighs outcome utility. While this study was designed to shift both components, emotional responses may extend beyond task-specific reactions. Although not originally hypothesized, we included mood as an exploratory variable, given its known sensitivity to Affect Labeling interventions [22] and its potential to shape task-related motivation more broadly. Whereas task aversion reflects specific emotional and cognitive evaluations of the task (e.g., boredom, dread, perceived difficulty), mood represents a more general affective state. By analyzing both the utility–aversion gap and mood as potential mediators, we aimed to more comprehensively capture the emotional and motivational pathways influenced by the intervention.

Empirical evidence supports interventions that reduce task aversion by aiming at specific negative attitudes or emotions. Examples include Cognitive Behavioral Therapy (CBT) [38, 57], Acceptance and Commitment Therapy (ACT) [9], and emotion regulation strategies [15, 40],each have demonstrated efficacy in reducing procrastination by enhancing emotional regulation skills. These interventions often employ self-monitoring and self-reflection techniques to identify and reframe maladaptive thoughts into goal-directed cognitions [30]. Additionally, time management and self-regulation strategies facilitate the translation of adaptive thoughts into behavior change [4, 37, 57]. However, these approaches often require significant time and effort from both participants and facilitators, making them resource-intensive and potentially cost-prohibitive. Designing a simpler, cost-effective, and less time-consuming technique to combat task aversion is crucial moving forward.

Interventions aimed at enhancing the tasks’ outcome utility have shown promise. Evidence suggests that breaking down larger tasks into smaller, manageable subgoals increases perceived achievability and expectancy of success, enhancing motivation [2]. Research on goal pursuit indicates that setting proximal subgoals can enhance motivation and lead to more successful outcomes by increasing the value of each step of progress [16]. Patria and Laili [31] furthered this conjecture by showing that a program incorporating elements such as setting specific writing targets and providing social support led to a significant decrease in procrastination levels. Indeed, this strategy of setting subgoals has been linked to increased volunteering behavior [33], and intrinsic motivation and task performance [52], underscoring its effectiveness in mitigating procrastination.

However, existing interventions predominantly involve longitudinal approaches and assess procrastination using dispositional measures, which capture generalized tendencies rather than situational behaviors. A meta-analysis of procrastination interventions revealed intervention durations ranging from 60 to 1,440 min (*M* = 404, *SD* = 365), with no significant moderation effect of intervention length on outcomes [59]. Additionally, procrastination was primarily measured as a dispositional trait, with limited focus on state-level procrastination.

While most procrastination interventions to date have focused on trait-level tendencies, i.e., stable patterns of delay that persist across time, such approaches often require intensive, long-term engagement and may not address the momentary decisions that give rise to procrastination in everyday life. Trait-based interventions, though valuable, are limited in their ability to offer immediate support when procrastination is most likely to occur: in the moment of task avoidance. In contrast, state-level procrastination refers to transient, situational delays influenced by task-specific factors and emotional states. Intervening at the state level offers the opportunity to influence decision-making processes in real time. The present study contributes to this emerging area by testing a brief, low-effort intervention designed to target these in-the-moment appraisals that lead to procrastination.

State procrastination refers to the tendency to delay tasks in the moment, influenced by situational factors such as task difficulty, emotional state, and immediate task aversiveness (e.g., [19]). Evidence suggests that it fluctuates across contexts and is more sensitive to short-term interventions (e.g., [20, 46]), such as the use of implementation intentions or real-time self-regulation techniques [13, 53]. The current research adds to the small but building literature on interventions used to tackle state-level procrastination.

Given the resource-intensive nature of longitudinal interventions and the gap in addressing momentary procrastination, the present study aims to develop a brief, cost-effective, and scalable intervention targeting state procrastination. By seeking to simultaneously reduce task aversion and enhance outcome utility, this study investigates whether a brief cognitive intervention can increase participants’ intention to act on a currently procrastinated task, which is a key proximal outcome relevant to real-time task engagement.

## The present study

With these considerations in mind, the current study 1) employs Affect Labeling to reduce task aversion and 2) time management and subtask generation to increase task outcome utility.

Affect Labeling, or the articulation of emotions into words, has been shown to reduce emotional reactivity to distressing stimuli [7, 11, 22, 58]. The most common method to employ Affect Labeling involves having participants choose, speak, or write a word that describes their feelings about the task at hand. In the present study, participants were prompted to contemplate and articulate the reasons behind their procrastination, leveraging this implicit emotion regulation strategy to alleviate distress. We anticipated that participants would write about emotions such as distress, frustration, anger, hopelessness, or boredom related to the task, thereby employing the Affect Labeling technique to reduce distress. We expected that this technique would result in participants reporting lower levels of feeling averse to doing the task, experiencing a more pleasant mood, and feeling less stressed compared to participants in the control groups.

Our second aim was to increase outcome utility by guiding participants through time estimation and subtask generation. Participants were instructed to select an easy subtask and estimate the time required to complete it. We conjectured that the easy subtasks would take less time to complete than the overall task, making the subtask’s outcome utility more immediate and attainable compared to that of the main task. Essentially, this process creates a pseudo-deadline that is closer and more manageable than the original deadline. Participants were also asked to identify a small reward they would receive upon completing the subtask. This introduces an extrinsic motivator and shifts the perceived value of goal completion closer to the present. Since the subtask requires less time to complete than the main task, this strategy effectively enhances the outcome utility of the task. We hypothesized that participants in the experiment group would thereby report greater outcome utility, and a higher motivation to complete the task than participants in the control groups.

We also hypothesized that, by combining Affect Labeling to reduce task aversion with subgoal generation and reward selection to enhance outcome utility, participants would exhibit an increased gap favoring outcome utility over aversion. Accordingly, this outweighing of outcome utility compared to task aversion should mediate the relationship between group assignment and the self-reported likelihood of completing the procrastinated task, consistent with cost–benefit decision-making models of procrastination.

We also sought to examine the impact of social desirability bias—“the tendency to give answers that make the respondent look good” [32], p. 17)—as a significant determinant of variables related to procrastination. Procrastination as a field largely relies on self-report data that is susceptible to self-reporting biases. Social desirability bias is more likely to occur in situations where behaviors, norms, and attitudes align with widely accepted standards or involve sensitive issues [14]. When responding to procrastination-related questions, participants may obscure true perceptions of their tasks, motivations, and intentions in order to present themselves favorably to the experimenter,this would substantially bias responses in self-report surveys. In the context of our study, this may result in participants overstating their likelihood of task completion, along with similar trends across other dependent variables. To explore the social desirability bias as a main predictor and/or moderator of effects on our dependent measures, we included a short form version of the Marlowe-Crowne Social Desirability Scale [60],see also [27].

In summary, this study takes an important first step in designing a brief, scalable intervention aimed at reducing state procrastination. The intervention integrates three evidence-based strategies—Affect Labeling, subgoal generation, and reward selection—to maximize its potential impact on self-reported task completion likelihood. By increasing perceived task utility and reducing task aversion, we hypothesized that participants in the experimental condition would report a greater intention to complete their procrastinated task compared to those in the control conditions.

## Method

### Participants and design

A total of 1,035 participants were recruited through Prolific. The sample size was determined by budget constraints, with each participant receiving $1 for completing the study. All participants completed a seriousness check, which asked them to indicate whether they had responded attentively or simply clicked through the survey. The prompt reassured participants that they would be compensated regardless of their answer to encourage honesty. Participants who reported not engaging seriously (*N* = 1) were excluded from the analysis [1]. The mean participant age was 38.99 years (*SD* = 13.63), and 63.3% identified as female. The racial composition of the sample was Asian = 7.5%, Black = 10.9%, White = 73.1%, Mixed = 4.9%, and Other = 3.6%, with all participants residing either in the United States of America or United Kingdom. Additionally, 14% of the sample consisted of students. This makes the study generalizable to broader adult populations, and the intervention scalable not just for academic but also general procrastination. On average, participants took 6.47 min (*SD* = 4 min) to complete the study. This study was approved by the University of California, Santa Barbara Human Subjects Committee (234–24-0776).

The study employed a single-factor between-subjects design with participants randomly assigned to one of three groups: the experimental group (*N* = 348), control group 1 (*N* = 336), or control group 2 (*N* = 351).

### Procedure

Participants on Prolific (www.prolific.com) first acknowledged a warning that the study would consist of multiple open-ended questions, and that they would not terminate their participation due to this [65]. Informed consent to participate was then obtained from all of the participants in the study. Then, participants were randomly assigned to one of the three groups.

Participants in the experiment group responded to 7 questions related to task specifics. The questions were all open-ended, requiring either one word or very brief responses. Participants were asked to name and describe one task they had been actively procrastinating. They were then asked to reflect on the qualities of the task, particularly their aversion to the task. Afterwards, they responded to a question asking about the perceived benefits of completing the task. Upon answering 4 questions related to the aforementioned, participants then named a subtask they could complete, followed by addressing the amount of time it would take them and indicating how they would reward themselves for completing the subtask.

Participants in control group 1 answered seven neutral, task-related questions that were structurally similar to those in the experimental condition (e.g., task location, who assigned the task), but lacked any affective or motivational content. These items were designed to control for the effects of time on task and engagement with the topic, without targeting task aversion or outcome utility. Participants in control group 2 were simply asked to name the task they are procrastinating (Table 1 lists the questions each condition was shown).

**Table 1:** Intervention and control conditions items

Experimental condition	Control condition (merged)	
	*Control 1*	*Control 2*
What task are you currently procrastinating on?	What task are you currently procrastinating on?	What task are you currently procrastinating on?
Provide a brief description of the task	Provide a brief description of the task	
Why are you avoiding doing this task?	What items do you need to complete the task?	
What are the benefits of completing this task?	What is the due date for this task?	
Tasks can be broken down into subtasks. Name an easy subtask you can complete for this task	When were you assigned this task?	
How long will it take you to complete this subtask (in minutes)?	Who assigned this task to you?	
Name a small reward for completing the subtask	What location does this task have to be completed in?	

Immediately after all conditions, participants rated the impact of the intervention on 6 dependent variables. 2 questions were adapted from Zhang et al. [63], where participants rated the outcome value, “How desirable are the benefits of completing this task?” (0 = extremely undesirable to 9 = extremely desirable) and task aversiveness, “How averse are you going to feel if you have to start or complete this task in the next 24 h?” (0 = extremely averse to 9 = extremely unaverse) of the task. 2 questions were related to their emotions upon having done the interventions, specifically inquiring about mood (0 = extremely unpleasant to 9 = extremely pleasant) and stress levels (0 = extremely stressed to 9 = extremely calm). Finally, participants rated their motivation to (0 = extremely unmotivated to 9 = extremely motivated) and likelihood of completing the task (0 = extremely unlikely, 9 = extremely likely). For the complete list of items, please refer to Table 2.

**Table 2:** Dependent variables list, with the preceding instructions stating: “The following items ask about the task you’ve been procrastinating on. Please respond to these questions.”

Variable name	Question	Likert range (9 point)
Task completion likelihood	In the next 24 h, how likely would you be to do this task?	Extremely unlikely—extremely likely
Outcome utility	How desirable are the benefits of completing this task?	Extremely undesirable—extremely desirable
Task aversion	How averse are you going to feel if you have to start or complete this task in the next 24 h?	Extremely averse—extremely unaverse
Mood	How would you rate your mood regarding this task?	Extremely unpleasant—extremely pleasant
Stress	How stressed do you feel regarding this task?	Extremely stressed—extremely calm
Motivation	How motivated do you feel to complete this task?	Extremely unmotivated—extremely motivated

Finally, all participants responded to the Brief Marlowe-Crowne Social Desirability Scale. This 10-item scale measures social desirability bias, or the tendency to present oneself in a socially desirable manner [60]. All items are measured using True/False statements, with an example item being “I have never intensely disliked anyone”. A high score suggests a tendency to appear more socially desirable.

Participants were then directed to answer the seriousness check question. This question enquires about how seriously the participant answered the questions in the study, with participants indicating less than ideal data being dropped from the analysis. All participants were given monetary compensation upon completion of the entire study.

## Results

### Analysis strategy

The study included two control groups, control group 1 and control group 2. Control group 1 was matched to the experimental group on the total number of items and asked neutral task-related questions, while control group 2 was simply asked to answer with a task they were procrastinating. This design of control groups enabled us to check if the presence of basic task-related questions would result in any difference from a condition where the participant only lists the task. If there was no difference, we could treat both control groups as an equivalent baseline condition. One-way ANOVAs across all groups were conducted, revealing no significant differences between the control groups in pairwise comparisons on any of the dependent variables.

To further reinforce a lack of significant differences between the control groups on all metrics, we also assessed the strength of evidence for the null hypothesis relative to the alternative examining Bayes Factors (BF₁₀) using the package *BayesFactor* [26]. Unlike traditional frequentist testing, Bayes Factors compare model strength rather than relying solely on *p*-values [8, 39]. Results consistently indicated moderate to strong evidence favoring the null hypothesis: BF₁₀ values between 0.01–0.03 reflect "very strong" evidence, 0.03–0.10 indicate "strong" evidence, and 0.10–0.33 suggest "moderate" evidence against the alternative hypothesis [61], p. 5, [17, 21, 39]. See Table 3 for detailed ANOVA and Bayes Factor results. Based on this, we merged the two control groups into one before testing our main hypotheses.

**Table 3:** Pairwise comparisons contrasts between the control groups, and the associated Bayes factor values

Dependent variable	Mean difference (95% CI)	*p*-value	Bayes Factor	Evidence strength
Outcome utility	−0.1442 (−0.447, 0.158)	0.503	0.1471 ± 0.13%	Moderate
Task aversion	−0.1149 (−0.463, 0.233)	0.718	0.1138 ± 0.17%	Strong
Utility–aversion	−0.0293 (−0.521, 0.462)	0.989	0.0859 ± 0.22%	Strong
Mood	−0.0690 (−0.369, 0.231)	0.851	0.0988 ± 0.19%	Strong
Stress	0.0061 (−0.334, 0.346)	0.999	0.0852 ± 0.22%	Strong
Motivation	−0.1011 (−0.463, 0.261)	0.789	0.1052 ± 0.18%	Strong
Task completion likelihood	−0.1462 (−0.591, 0.299)	0.721	0.1137 ± 0.17%	Strong

### Group differences

To assess whether the experimental group differed significantly from the control group, Welch’s two-sample t-tests were conducted on seven self-reported measures related to participants' procrastinated tasks.

#### Task completion likelihood

**Fig. 1:**
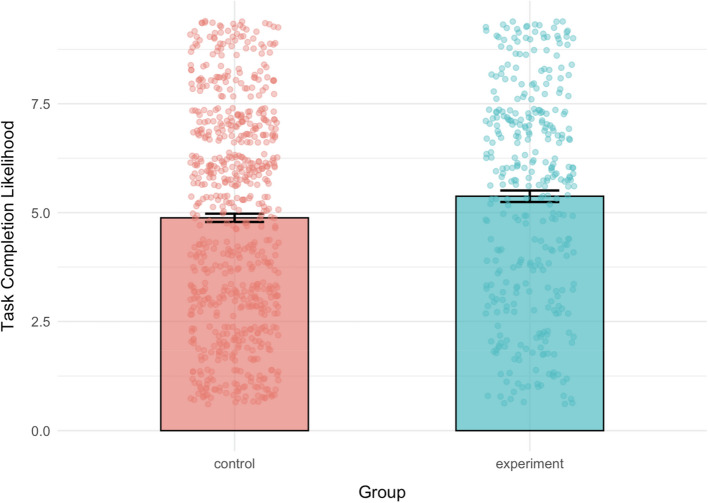
Mean of reported likelihood of task completion on a 9-point likert scale for each group

The reported likelihood of completing the procrastinated task in the next 24 h was significantly higher for the experimental group (*M* = 5.38, *SD* = 2.47) than for the control group (*M* = 4.88, *SD* = 2.49), *t*(703.98) = −3.04, *p* = 0.002, 95% CI [−0.82, −0.18], *d* = −0.2 (Fig. 1).

#### Outcome utility

**Fig. 2:**
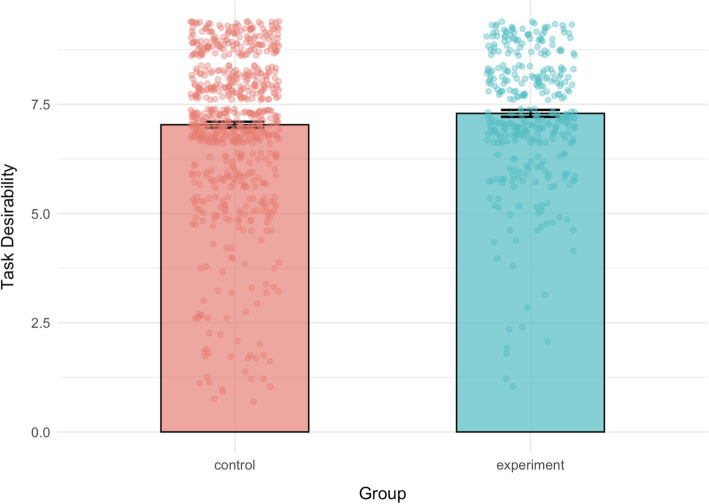
Mean of outcome utility of task completion on a 9-point likert scale for each group

The outcomes of the task were reported as more desirable by the participants in the experimental group (*M* = 7.29, *SD* = 1.47) than the control group (*M* = 7.03, *SD* = 1.79), *t*(824.95) = −2.49, *p* = 0.013, 95% CI [−0.46, −0.05], *d* = −0.15 (Fig. 2).

#### Task aversion

The results indicated no significant difference between the experimental group (*M* = 4.42, *SD* = 1.91) and the control group (*M* = 4.56, *SD* = 1.95) on how averse participants felt about completing the task in the near future, *t*(710.29) = 1.11, *p* = 0.267, 95% CI [−0.11, 0.39], *d* = 0.07, 95% CI [−0.06, 0.20].

#### Utility–aversion

**Fig. 3:**
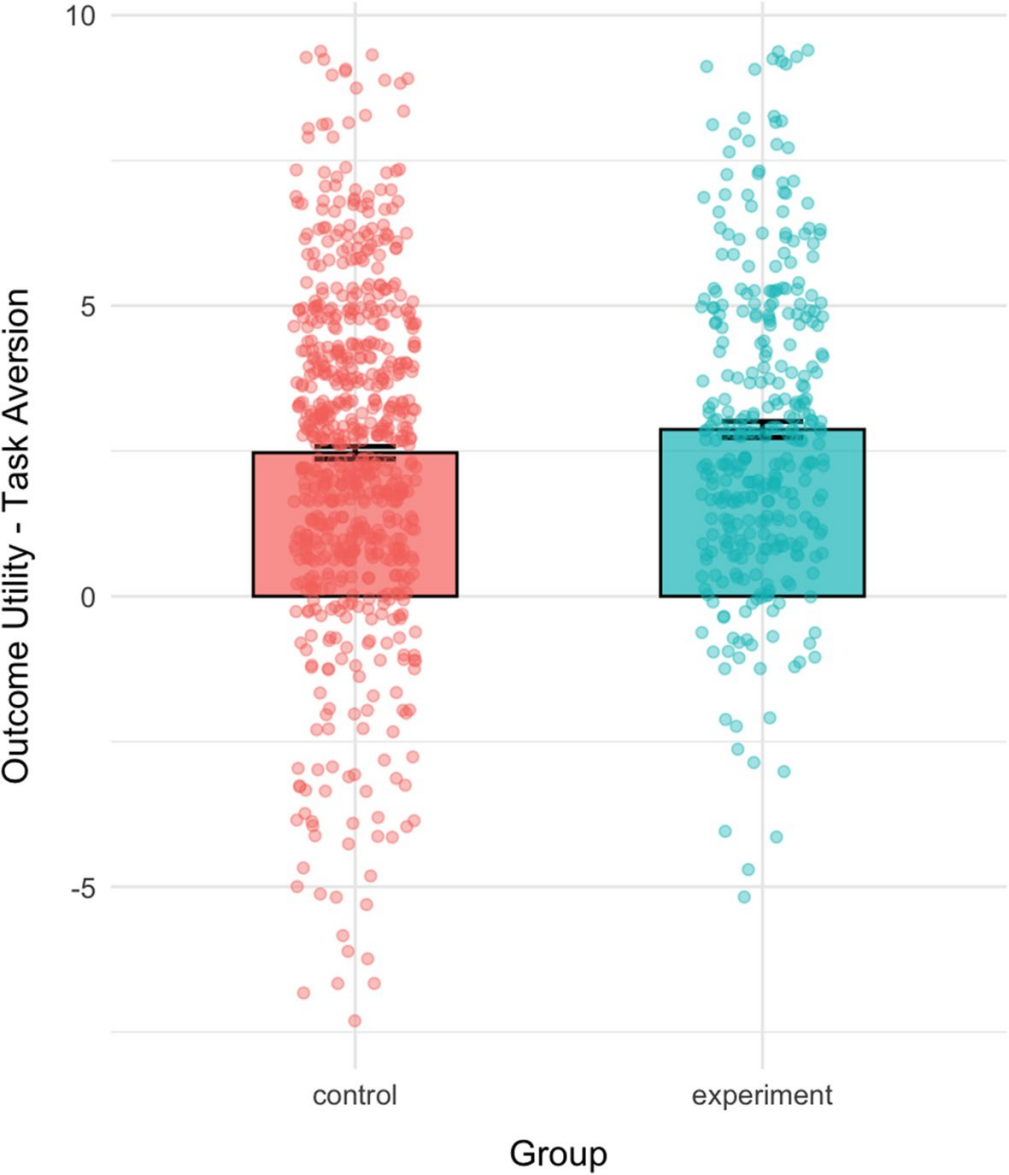
Mean of the gap between outcome utility and task aversion, calculated as the difference between the two, for each group

We computed the difference between outcome utility and task aversion to create a cost–benefit balance score, where a positive number indicates that outcome utility outweighs task aversion. The results suggested a significant difference between the groups, such that the participants in the experimental group (*M* = 2.87, *SD* = 2.57) reported a larger difference between outcome utility and task aversion than participants in the control group (*M* = 2.47, *SD* = 2.82), *t*(757.29) = −2.29, *p* = 0.022, 95% CI [−0.74, −0.06], *d* = −0.15 (Fig. 3).

#### Mood

**Fig. 4:**
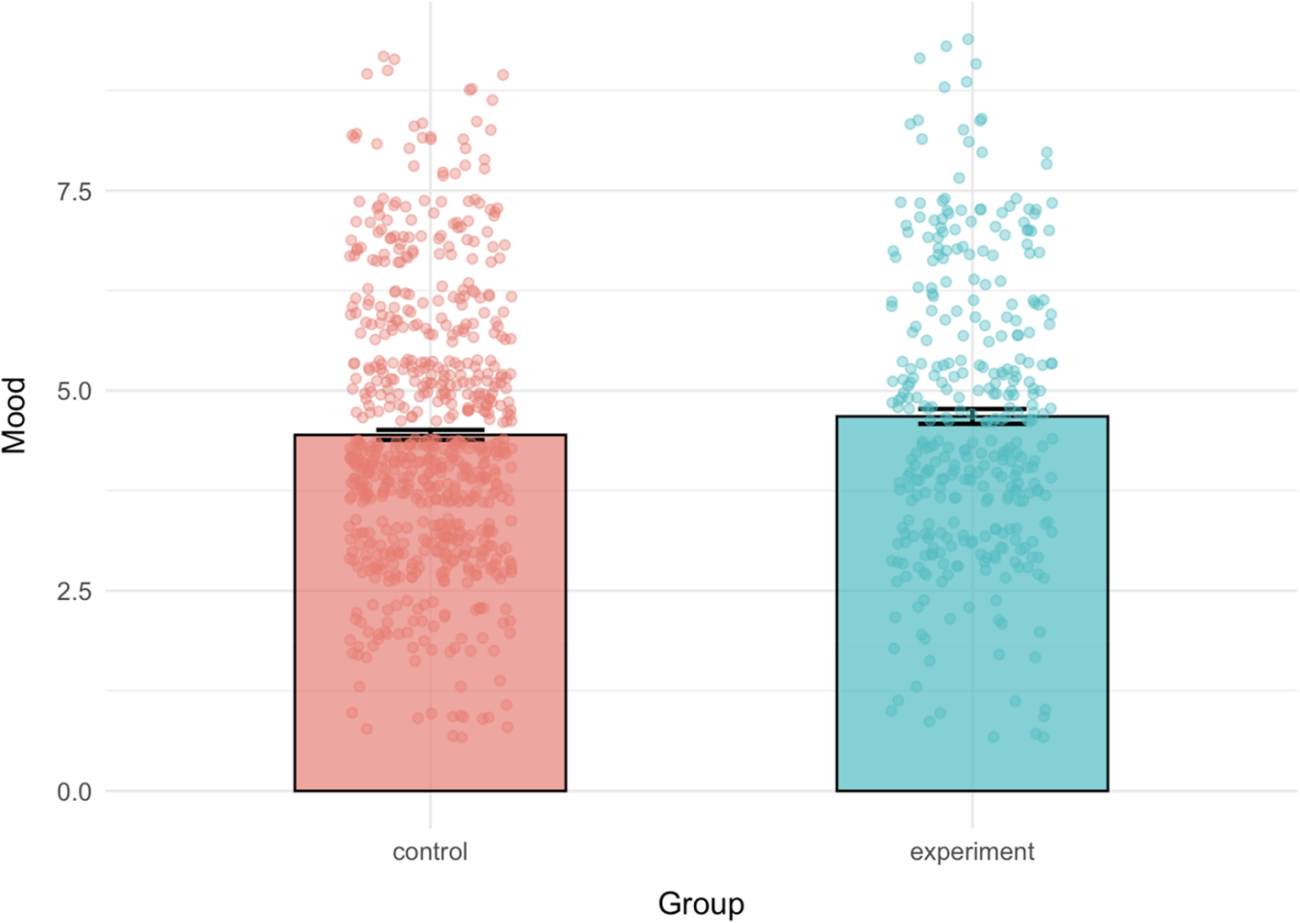
Mean of reported mood on a 9-point likert scale (higher values indicate a more pleasant mood) for each group

Participants in the experimental group (*M* = 4.68, *SD* = 1.73) reported having a significantly more pleasant mood regarding the task compared to participants in the control group (*M* = 4.45, *SD* = 1.64), *t*(664.59) = −2.06, *p* = 0.039, 95% CI [−0.45, −0.01], *d* = −0.14 (Fig. 4).

#### Stress

The results indicated no significant difference between the experimental group (*M* = 4.52, *SD* = 1.94) and the control group (*M* = 4.73, *SD* = 1.88) regarding the stress related to the task, *t*(675.90) = 1.66, *p* = 0.098, 95% CI [−0.04, 0.46], *d* = 0.11.

#### Motivation

The results indicated no significant difference in the motivation to complete the task between the experimental group (*M* = 4.59, *SD* = 2.03) and the control group (*M* = 4.38, *SD* = 2.01), *t*(692.13) = −1.59, *p* = 0.112, 95% CI [−0.47, 0.05],* d* = −0.11.

### Mediation analysis

Given that both the outcome utility-task aversion difference and mood were found to be significant predictors of task completion likelihood, we conducted parallel mediation analysis using *lavaan* [36] to examine whether the effect of group assignment (experimental vs. control) on task completion likelihood was mediated by outcome utility-task aversion difference and mood.^1^ This model was saturated, and therefore fit the data perfectly by definition.

The direct effect of group assignment on task completion likelihood was significant, *b* = 0.34, *SE* = 0.15, *z* = 2.20, *p* = 0.028, 95% CI [0.04, 0.63], reaffirming that participants in the experimental group reported a higher likelihood of completing their procrastinated task compared to the control group.

Regarding the mediators, group assignment significantly predicted both the utility–aversion difference (*b* = 0.40, *SE* = 0.18, *z* = 2.26, *p* = 0.024, 95% CI [0.06, 0.75]) and mood (*b* = 0.23, *SE* = 0.11, *z* = 2.07, *p* = 0.039, 95% CI [0.01, 0.45]). In turn, both the utility–aversion difference (*b* = 0.21, *SE* = 0.03, *z* = 6.90, *p* < 0.001, 95% CI [0.15, 0.27]) and mood (*b* = 0.33, *SE* = 0.05, *z* = 7.00, *p* < 0.001, 95% CI [0.24, 0.42]) were significant predictors of task completion likelihood.

The indirect effect of group assignment on task completion likelihood through the utility–aversion difference was significant, *b* = 0.09, *SE* = 0.04, *z* = 2.16, *p* = 0.031, 95% CI [0.01, 0.16]. The indirect effect through mood approached significance, *b* = 0.08, *SE* = 0.04, *z* = 1.94, *p* = 0.053, 95% CI [0.00, 0.16]. The total indirect effect was significant, *b* = 0.16, *SE* = 0.06, *z* = 2.61, *p* = 0.009, 95% CI [0.04, 0.28], indicating that the combined influence of both mediators accounted for a significant portion of the relationship between group assignment and task completion likelihood.

**Fig. 5:**
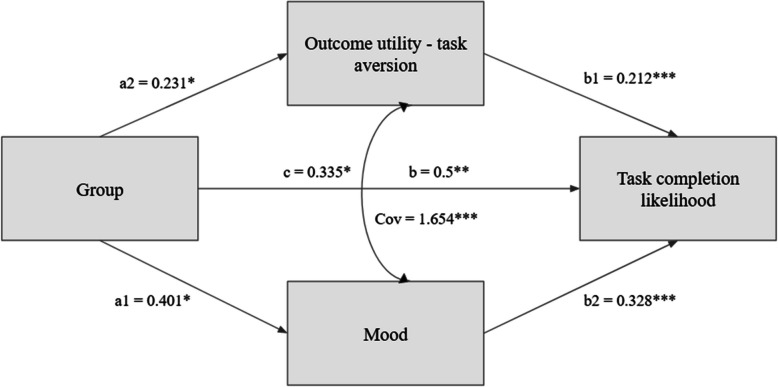
Mediation model of group differences being mediated by mood and utility–aversion on task completion likelihood. Indirect effects are shown as paths from group assignment through utility–aversion (a1 × b1, *b*: 0.09) and mood (a2 × b2, *b*: 0.08) to task completion likelihood, combining into a total indirect effect of 0.16. The direct effect (c = 0.34) and total effect (b = c + total indirect effect = 0.50) are also depicted. Please note that *p <.05, **p <.01, ***p <.001

Finally, the total effect of group assignment on task completion likelihood was significant, *b* = 0.50, *SE* = 0.16, *z* = 3.03, *p* = 0.002, 95% CI [0.17, 0.81], suggesting that both direct and indirect pathways contributed to the observed increase in task completion likelihood in the experimental group (Fig. 5).

### Social desirability bias

To assess whether the observed group differences on task-related outcomes were influenced by social desirability bias, a series of ANCOVAs were conducted with the Marlowe-Crowne Social Desirability Scale (MCSD) included as a covariate. The pattern of results remained consistent, with group assignment predicting task completion likelihood, outcome utility, and mood even after adjusting for social desirability. Full ANCOVA results are reported below.

The analysis revealed significant main effects of group assignment on likelihood of task completion, *F*(1, 1031) = 9.20, *p* = 0.002, outcome utility, *F*(1, 1031) = 5.45, *p* = 0.020, utility–aversion difference, *F*(1, 1031) = 4.93, *p* = 0.027, and mood, *F*(1, 1031) = 4.59, *p* = 0.032.. These results suggest that the experimental group reported a higher likelihood of completing the task, greater outcome utility, a larger gap between outcome utility and task aversion, and a more pleasant mood compared to the control group, even after accounting for social desirability bias.

**Fig. 6:**
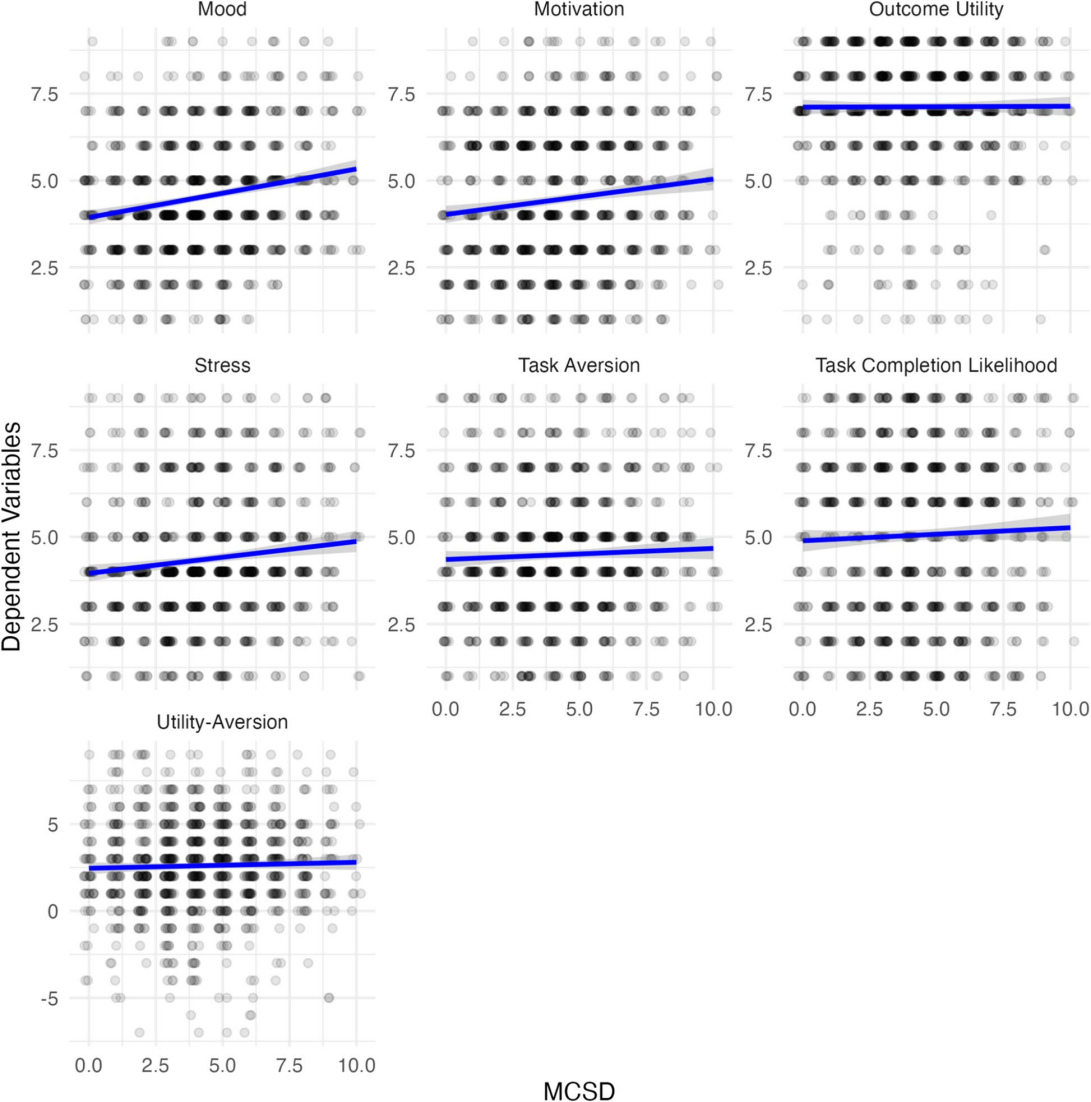
Scatter plots of social desirability and the 7 dependent variables: mood, motivation, outcome utility, task aversion, utility–aversion, stress, and task completion likelihood

In contrast, no significant group differences emerged for task aversion, *F*(1, 1031) = 1.22, *p* = 0.270, stress, *F*(1, 1031) = 2.84, *p* = 0.092, or motivation, *F*(1, 1031) = 2.58, *p* = 0.108. Additionally, the main effect of MCSD and the interaction between group and MCSD were non-significant across all models (*ps* > 0.22), indicating that social desirability bias did not meaningfully influence the observed outcomes. Exploratory scatterplots depicting the bivariate relationships between MCSD and each outcome variable are presented in Fig. 6.

## Discussion

Procrastination is a pervasive and maladaptive behavior characterized by the irrational delay of tasks, often resulting in adverse personal and professional consequences. Addressing this behavior through effective interventions is critical. The present study aimed to develop and evaluate a brief, low-effort intervention grounded in the Temporal Decision Model [62], which integrates emotion regulation and temporal motivation perspectives to conceptualize procrastination as the result of a dynamic cost–benefit analysis between outcome utility and task aversion. By targeting these components, the intervention sought to shift appraisals associated with state procrastination by increasing outcome utility and decreasing task aversion. Although the intervention did not measure actual behavior, participants reported a greater intention to complete their procrastinated task, suggesting that brief strategies may support goal-related decision making in moments of delay.

The findings of this study provide empirical support for the intervention's effectiveness. Participants who completed the intervention, which consisted of seven questions that guided respondents through Affect Labeling, subgoal generation, and reward selection techniques, reported significantly greater outcome utility, a larger gap favoring outcome utility over task aversion, more positive mood states, and a higher self-reported likelihood of task completion compared to the control group. These results suggest that brief, targeted interventions may help influence the processes that contribute to state procrastination—particularly by enhancing perceived outcome utility relative to task aversion—which may, in turn, increase individuals’ intentions to follow through on delayed tasks.

Notably, the results indicate that increasing the perceived outcome utility of a task relative to its aversiveness positively influences individuals' reported likelihood of completing procrastinated tasks. Specifically, when participants perceived the desirability of task outcomes as outweighing the aversiveness of task engagement, they were more likely to report intentions to complete the task in the near future. This finding aligns with the Temporal Decision Model, which posits that procrastination persists until outcome utility surpasses task aversion as a deadline approaches [62, 64]. Importantly, even without significantly reducing task aversion, enhancing outcome utility was sufficient to increase participants' reported intention to act.

These findings suggest that the intervention may have influenced participants' task-related decision-making through multiple psychological pathways. The utility–aversion gap is directly aligned with the TDM’s framework of cost–benefit evaluations, while mood likely captures broader emotional effects of the Affect Labeling component. Though mood was not a pre-registered mediator, its inclusion is theoretically grounded in prior work showing that labeling negative emotions can reduce general distress [22]. Given that task aversion did not significantly differ between groups, the item used to assess it may have captured more cognitive or evaluative components of the task (e.g., difficulty, effort, boredom) than emotional aversion. As such, mood may have absorbed some of the emotional variance not captured by our task-specific aversion item. Future work may benefit from more granular emotion measures to distinguish between general and task-specific emotional responses.

This outcome underscores the potential of interventions that elevate outcome utility, particularly by introducing extrinsic motivators such as small rewards for completing subgoals. Such strategies can shorten the psychological distance between task engagement and goal realization, thereby increasing the perceived value of task completion in the present. Given that state procrastination reflects a momentary, context-dependent reluctance to engage with a task, interventions that shift the immediate cost–benefit appraisal (by enhancing utility or reducing aversion) appear well-suited to disrupting this temporary state of inaction. By targeting state procrastination directly, brief interventions may offer timely support when individuals are most vulnerable to task avoidance. However, it is important to note that the mediation effect of the utility–aversion gap was partial, suggesting that the effect of the intervention on task completion likelihood is not entirely explained by the change in task utility–aversion.

While outcome utility and mood partially mediated the effect of the intervention on self-reported task completion likelihood, a significant portion of the total effect remained unexplained. This residual effect suggests that other psychological mechanisms may have contributed to the intervention’s influence on participants' motivation and intention to act. One promising framework for interpreting this variance is Temporal Motivation Theory (TMT) [50], which integrates expectancy theory, hyperbolic discounting, and need theory. According to TMT, motivation is not solely a function of perceived task value (i.e., outcome utility) but is also shaped by delay sensitivity (i.e., impulsiveness), expectancy of reward achievement, and temporal proximity to reward. Because our intervention prompted participants to break down the task and visualize near-term benefits, it may have altered their temporal framing of the task, reducing the perceived delay between effort and reward. This shift could be especially motivating for individuals high in impulsiveness, who tend to discount distant rewards more steeply [35, 50]. Additionally, by encouraging participants to choose a small reward for completing a subtask, the intervention may have increased self-efficacy by creating a sense of control and reinforcing participants’ belief in their ability to take meaningful action toward task completion [18]. Research has shown that proximal, achievable goals paired with contingent rewards can strengthen self-efficacy beliefs, which in turn enhance motivation and persistence [3]. Future research examining additional potential mediators could help enhance the efficacy of this intervention and increase its robustness.

Another potential mechanism is learned industriousness, the idea that effort can become rewarding when consistently paired with reinforcement (Eisenberger, 1992). By encouraging subgoal planning and associated rewards, the intervention may have temporarily reactivated this effort–reward association, boosting motivation beyond shifts in utility or mood. While not directly tested here, future work could explore whether brief interventions like this one help build lasting motivation by reinforcing effort itself.

Interestingly, the intervention did not significantly impact task aversiveness. This may be due to two reasons: 1) the administration method of the technique was not explicit, or 2) the Affect Labeling technique was not robust enough to effectively reduce distress among participants. On the first point, participants were not explicitly instructed to label their emotions. When asked to reflect on reasons for task avoidance, many participants cited task-related characteristics (e.g., boring, difficult, lengthy) rather than emotional responses. This aligns with literature suggesting that task aversiveness stems from both individual and task characteristics [48, 49]. Explicitly directing participants to label their emotions may enhance the efficacy of Affect Labeling in reducing task aversion.

Second, prior research on Affect Labeling has yielded mixed results. For example, Ortner [29] reported that Affect Labeling increased self-reported distress without affecting physiological arousal, whereas Matejka et al. [24] observed decreased autonomic responses but noted that factual discussions reduced self-reported emotional intensity more effectively. Conversely, Burklund et al. [6] demonstrated that Affect Labeling reduced distress, paralleling effects seen with cognitive reappraisal. Despite these inconsistencies, the current study found that participants in the intervention group reported significantly more positive mood states than controls. This suggests that even in its minimal, non-explicit form, Affect Labeling may have contributed meaningfully to emotional regulation. Future research should continue exploring Affect Labeling as a component of brief procrastination interventions, potentially in combination with other strategies like mindfulness, cognitive reappraisal, or brief CBT (e.g., [34]), to enhance its impact on both emotion and motivation.

Our final finding was that our procrastination-related dependent variables were mostly nonreactive to social desirability bias. Results indicated that social desirability bias did not interact with group assignment on any dependent variables, though it had a main effect on mood, stress, and motivation. This finding suggests that the observed improvements in outcome utility, mood, and self-reported likelihood of task completion are not artifacts of participants responding in socially desirable ways. We argue that this reinforces the validity of the present findings. While our results presented here also suggest that social desirability bias has not confounded much of the results elsewhere in the procrastination literature, we advise other researchers to control for social desirability bias to strengthen the credibility of self-report-based procrastination studies.

### Limitations

The current study is not without its limitations. First, actual task completion rates were not measured. Instead, participants self-reported their likelihood of completing the procrastinated task within the next 24 h. This reliance on self-reports raises concerns related to the intention-behavior gap, which highlights discrepancies between individuals' stated intentions and their subsequent actions [41]. Self-regulation capabilities, a key moderating factor in this gap, have also been identified as a critical individual difference influencing procrastination. Consequently, the present findings suggest that the intervention increased participants' intentions to complete the task but do not conclusively indicate actual task completion. Future research should address this limitation by examining the intention-action gap in procrastination. Specifically, employing longitudinal or two-part study designs where participants are followed up or recalled to report actual task completion, would provide more robust evidence of the intervention's effectiveness.

Second, the current study utilized a combined intervention targeting both outcome utility and task aversion simultaneously. It is uncertain which specific component of the intervention—subgoal generation, the introduction of a small reward, or the recall of task benefits—was the most influential in driving the observed improvements in outcome utility, mood, and reported likelihood of task completion. Future research should systematically evaluate each intervention component independently to identify which strategy exerts the greatest impact on reducing procrastination.

Finally, although many results of the present study were statistically significant, the observed effect sizes were relatively small. This raises concerns about the practical significance of the findings and risks overinterpretation. The modest effect sizes suggest that while the intervention may be effective, its impact compared to the control group is limited. Replication of the current study, along with extensions incorporating more robust and explicit interventions designed to further enhance outcome utility and reduce task aversion, is recommended to strengthen the evidence base and maximize practical relevance.

## Implications and conclusion

Developing cost-effective, low-threshold interventions to address procrastination is an urgent need, given the substantial negative impact of procrastination on individuals' well-being and functioning. Overcoming barriers such as limited treatment access, long wait times, and the demands of intensive therapy is crucial for widespread intervention adoption. Although some interventions have been designed to target procrastination, there is a scarcity of research on task-specific interventions validated through rigorous randomized controlled trials (see [38, 59]).

The current study makes a meaningful contribution by demonstrating that a brief, low-effort intervention can effectively enhance outcome utility and increase the likelihood of task completion. By leveraging the Temporal Decision Model, this work provides valuable insights into how balancing task aversion and outcome utility can influence intentions to act upon procrastinated tasks.

Practical implications to combating procrastination in educational, workplace, and clinical settings could utilize internet- and mobile-based interventions (see [10, 12, 23, 37, 38]). Digital platforms and mobile applications could integrate brief, utility-enhancing strategies, such as gamification elements or personalized incentives, to encourage task initiation and completion. By focusing on increasing outcome utility rather than solely attempting to reduce task aversion, such interventions may offer an accessible and effective means to reduce procrastination and improve productivity across diverse populations. As such, these findings pave the way for developing scalable, accessible interventions to manage procrastination and offer a strong foundation for future research aimed at refining and optimizing strategies to support goal-directed action.

## Data Availability

Data available on request from the authors.

## Appendix A

### Mediation analysis using only the utility–aversion difference as a mediator

To maintain transparency with our pre-registered analytic plan, we present here the originally hypothesized mediation model, which included only the outcome utility–task aversion difference as a mediator of the relationship between group assignment and task completion likelihood.

Given the model was saturated, it fit the data perfectly by definition.

The direct effect of group assignment on task completion likelihood was significant, b = 0.38, SE = 0.15, *z* = 2.48, *p* = 0.013, 95% CI [0.07, 0.68], indicating that participants in the experimental group reported a higher likelihood of completing their procrastinated task compared to the control group.

Group assignment significantly predicted the utility–aversion difference, b = 0.40, SE = 0.17, *z* = 2.31, *p* = 0.021, 95% CI [0.06, 0.75], and the utility–aversion difference in turn significantly predicted task completion likelihood, b = 0.28, SE = 0.03, *z* = 9.99, *p* < 0.001, 95% CI [0.23, 0.34].

The indirect effect of group assignment on task completion likelihood through the utility–aversion difference was significant, b = 0.11, SE = 0.05, *z* = 2.29, *p* = 0.022, 95% CI [0.02, 0.21].

The total effect of group assignment on task completion likelihood remained significant, b = 0.50, SE = 0.16, *z* = 3.04, *p* = 0.002, 95% CI [0.17, 0.82], demonstrating that the utility–aversion difference accounts for a significant portion of the observed increase in task completion likelihood among participants in the experimental group.

## Footnotes

1 The original hypothesized mediation model, which includes only the utility–aversion gap as a mediator, is reported in Appendix A.
